# Multiscale Coupling of an Agent-Based Model of Tissue Fibrosis and a Logic-Based Model of Intracellular Signaling

**DOI:** 10.3389/fphys.2019.01481

**Published:** 2019-12-17

**Authors:** S. Michaela Rikard, Thomas L. Athey, Anders R. Nelson, Steven L. M. Christiansen, Jia-Jye Lee, Jeffrey W. Holmes, Shayn M. Peirce, Jeffrey J. Saucerman

**Affiliations:** ^1^Department of Biomedical Engineering, University of Virginia, Charlottesville, VA, United States; ^2^Department of Biomedical Engineering, Johns Hopkins University, Baltimore, MD, United States; ^3^Department of Pharmacology, University of Virginia, Charlottesville, VA, United States; ^4^Robert M. Berne Cardiovascular Research Center, University of Virginia, Charlottesville, VA, United States; ^5^Department of Medicine, University of Virginia, Charlottesville, VA, United States

**Keywords:** fibrosis, cardiac fibroblast, myocardial infarction, systems biology, multiscale modeling, agent-based model, network model

## Abstract

Wound healing and fibrosis following myocardial infarction (MI) is a dynamic process involving many cell types, extracellular matrix (ECM), and inflammatory cues. As both incidence and survival rates for MI increase, management of post-MI recovery and associated complications are an increasingly important focus. Complexity of the wound healing process and the need for improved therapeutics necessitate a better understanding of the biochemical cues that drive fibrosis. To study the progression of cardiac fibrosis across spatial and temporal scales, we developed a novel hybrid multiscale model that couples a logic-based differential equation (LDE) model of the fibroblast intracellular signaling network with an agent-based model (ABM) of multi-cellular tissue remodeling. The ABM computes information about cytokine and growth factor levels in the environment including TGFβ, TNFα, IL-1β, and IL-6, which are passed as inputs to the LDE model. The LDE model then computes the network signaling state of individual cardiac fibroblasts within the ABM. Based on the current network state, fibroblasts make decisions regarding cytokine secretion and deposition and degradation of collagen. Simulated fibroblasts respond dynamically to rapidly changing extracellular environments and contribute to spatial heterogeneity in model predicted fibrosis, which is governed by many parameters including cell density, cell migration speeds, and cytokine levels. Verification tests confirmed that predictions of the coupled model and network model alone were consistent in response to constant cytokine inputs and furthermore, a subset of coupled model predictions were validated with *in vitro* experiments with human cardiac fibroblasts. This multiscale framework for cardiac fibrosis will allow for systematic screening of the effects of molecular perturbations in fibroblast signaling on tissue-scale extracellular matrix composition and organization.

## Introduction

Approximately 605,000 Americans experience their first myocardial infarction (MI) each year, and another 200,000 experience a recurrent MI (Benjamin et al., 2018). Approximately 82% of males and 77% of females survive at least 1 year following their MI (Benjamin et al., 2018), making management of post-MI recovery an increasingly important topic.

Wound healing and scar remodeling following MI is a dynamic process involving many cell types, extracellular matrix, and inflammatory cues. Myocyte death due to prolonged ischemia initiates an inflammatory response led by cytokines such as IL-1β and TNFα (Frantz et al., 2009). Neutrophils and macrophages are recruited to the wound site within 24 h and begin to phagocytose debris and propagate the inflammatory response. Inflammatory cells peak within the first week of wound healing and then begin to subside as the proliferative phase begins (Czubryt, 2012). Inflammatory macrophages secrete TGFβ, which stimulates fibroblast recruitment and proliferation (Lambert et al., 2008). The release of TGFβ may also contribute to the conversion of macrophages to a more anti-inflammatory phenotype (Lambert et al., 2008). The proliferative phase may last for days to weeks and is marked by the proliferation of fibroblasts and transition to a myofibroblast phenotype, along with synthesis of many ECM components including collagen (Czubryt, 2012). ECM deposition produces a scar in the infarct region that contributes to its structural stability during wound healing. This proliferative phase is followed by weeks to months of scar remodeling and significant ECM turnover.

Post-MI cardiac wound healing is a complex and dynamic process with many overlapping phases. The cardiac fibroblast is the key effector cell throughout the phases of wound healing that creates and remodels scar tissue (Spinale et al., 2016; Mouton et al., 2019). However, fibroblasts are a highly dynamic and plastic cell type that can transition from a pro-inflammatory phenotype in the early phases of wound healing to an anti-inflammatory and pro-fibrotic phenotype later in the wound healing cascade (Chen and Frangogiannis, 2013; Mouton et al., 2019). Fibroblast response to single cytokine inputs are well-documented (Fredj et al., 2005; Fix et al., 2011; Turner, 2014), but fibroblast activation and cytokine secretion in response to multiple cytokines and other stimuli *in vivo* that shift over the time course of MI wound healing are not well-described (Ma et al., 2017). This lack of understanding of activation shifts over the time course of healing is at the core of the failure of many attempts to improve post-MI wound healing by modulating scar formation (Clarke et al., 2016). Inhibition of inflammation too early in the wound healing cascade can lead to thinning of the LV wall and scar rupture (Brown et al., 1983; Hammerman et al., 1983a,b). Aberrant fibrosis can lead to LV dilation and heart failure. This inherent complexity of the biological phenomenon necessitates the development of computational models to design and test therapeutic interventions that potentially have opposite effects at different phases throughout the wound healing cascade. Previous computational models have extensively characterized cardiac fibroblast signaling pathways and expression profiles to provide information about fibroblast activation and kinetics (Nim et al., 2015; Zeigler et al., 2016a,b), but fibroblast activation has generally been studied in response to single stimuli *in vitro*. Other researchers in the field have noted the need to understand fibroblast activation in response to mixed stimuli, and have called for the development of computational models that can integrate the effects of spatial and temporal shifts in fibroblast activation, with the cell-cell interactions and cell-matrix interactions that coordinate the short and long-term remodeling of scar tissue (Ma et al., 2017). A multiscale model that can translate cardiac fibroblast gene and protein expression to tissue level functional remodeling with spatial and temporal precision could provide an invaluable platform for identifying, testing, and validating new therapeutic interventions for inducing functional regeneration and mitigating fibrosis.

Our group has recently developed computational models to study distinct scales of cardiac wound healing, including a logic-based differential equation (LDE) model of intracellular signaling in individual cardiac fibroblasts and an agent-based model (ABM) of collagen remodeling by multiple cells in the infarct (Rouillard and Holmes, 2012; Zeigler et al., 2016a). Each model represents a different spatial and temporal scale of the wound healing process. The LDE model provides detailed information about the network state of 91 different signaling nodes in an individual fibroblast, while the ABM predicts fibroblast number, collagen area fraction, and collagen alignment at the tissue level. In the work presented here, we couple these LDE and ABM models in order to capture the dynamic interplay between fibroblast intracellular signaling and spatially heterogeneous extracellular cues such as cytokines and ECM composition, which themselves are modulated by individual fibroblast behaviors. Verification tests confirmed that the coupled model and network model alone exhibit consistent behavior in response to constant cytokine and growth factor inputs, allowing for the establishment of a framework that can readily incorporate updates from either the network model or ABM without affecting the integrity of the individual model predictions. Furthermore, a subset of coupled model predictions was validated by comparison to measurements of pro-collagen 1, αSMA, and F-actin expression in human cardiac fibroblasts treated with combinations of cytokines and growth factors *in vitro*. We believe this work demonstrates the first coupling of a large-scale network model to predict tissue-level changes in ECM composition in the setting of fibrosis with feedback from environmental cues (e.g., diffusible cytokines) to regulate the signaling of individual cells. Predictions about cytokine and growth factor production from fibroblasts are computed in physical units, which were not previously possible with a logic-based network model alone. This coupled model provides a platform for systematically testing molecular interventions with the ability to measure their effects on single cell signaling and ECM composition with detailed spatial resolution.

## Materials and Methods

### Description of Individual Models

#### Agent-Based Model

An agent-based model (ABM) is comprised of value layers and agents (An et al., 2009). The value layers in this two-dimensional ABM represent features of the extracellular space, including collagen, latent TGFβ, active TGFβ, IL-1β, IL-6, and TNFα. All cytokines are stored as concentrations in pg/mL, and collagen is quantified as an area fraction. The value layers are divided into a 10 × 10 grid, where each individual grid space measures 10 × 10 μm. A volume for each grid space is approximated based on cell culture conditions in a 96 well-plate, which is the primary source of experimental data used to inform this model. For soluble cytokines (active TGFβ, IL-1β, IL-6, and TNFα), it is assumed that these cytokines are uniformly distributed in the media above each cell, resulting in a compartment of 10 × 10 × 3125 μm (3.125e-7 mL). Latent TGFβ binds to the extracellular matrix (Horiguchi et al., 2012), and is thus assumed to occupy the space immediately surrounding the cell, or 10 × 10 × 10 μm (1e-9 mL). The individual grid space approximates the footprint of a single fibroblast, allowing the model to simulate a maximum of 100 fibroblasts simultaneously. The total number of fibroblasts is kept relatively low to allow for calculation of the entire network state of each fibroblast while minimizing computational time for the purposes of method development. Value layers store a unique quantity in each grid space that can be modulated by parameters including degradation rates, activation rates, and the agents that move over them. The agents in this model represent cardiac fibroblasts that migrate and modulate their extracellular space by depositing and degrading collagen, and secreting cytokines.

#### Logic-Based Network Model

The logic-based differential equation (LDE) network model is a previously published (Zeigler et al., 2016a) model of cardiac fibroblast signaling that integrates 10 signaling pathways with 11 biochemical or mechanical stimuli that are important for myofibroblast activation and ECM remodeling. These stimuli include IL-1 (interleukin 1), IL-6 (interleukin 6), TNFα (tissue necrosis factor α), NE (norepinephrine), NP (natriuretic peptide), β-integrins, TGFβ (tissue growth factor β), angiotensin II, PDGF (platelet derived growth factor), ET1 (endothelin 1), mechanical stimulation, and forskolin. The network includes 91 nodes connected by 142 reactions, which are supported by *in vitro* data collected from cardiac fibroblasts. The network was constructed using a logic-based ordinary differential equation modeling approach, where the activity of each node is modeled using a normalized Hill ODE with default parameters and logic gating. Default reaction parameters include weight (0.9), Hill coefficient (1.4), and EC_50_ (0.6), and species parameters include y_init_(0), y_max_(1), and τ. The τ parameter (time constant) was scaled according to the type of reaction: 6 min for signaling reactions, 1 h for transcription reactions, and 10 h for translation reactions. The baseline level of input is defined as 25% activity for all input nodes. The system of ODEs is generated using the Netflux software available at: https://github.com/saucermanlab/Netflux, and implemented in MATLAB.

### Coupled Model

#### Interactions That Drive the Coupled Model

**Figure 1:**
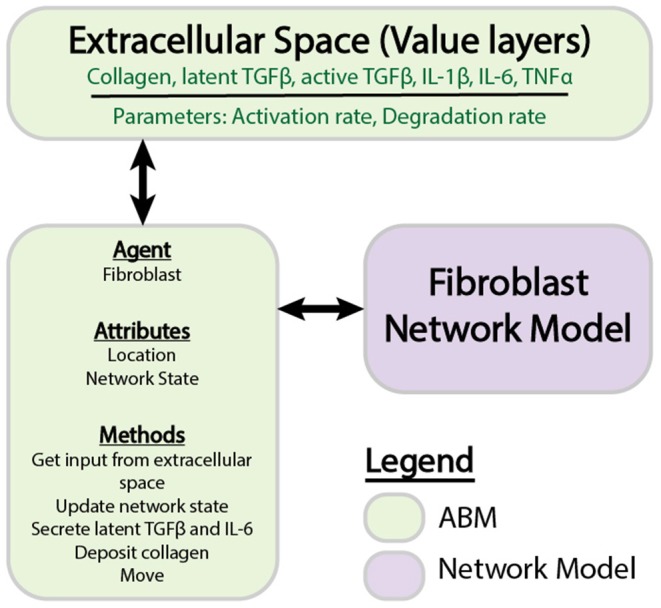
Components of individual ABM and network models. The ABM is comprised of agents that store information about attributes and perform methods. Value layers can be modified independently by defined parameters or by the activity of agents. Individual agents store a network state, which is updated by the fibroblast network model.

Figure 1 provides an overview of the components and interactions between the LDE network model and ABM. The ABM contains the value layers that represent the extracellular space and the cardiac fibroblasts that migrate over and interact with these value layers. The time step for this coupled model is 1 h, representing the approximate timescale for a change in input to the cell signaling network to affect production of cytokines and ECM proteins that will be deposited in the ABM (Enríquez-de-Salamanca et al., 2008; Azghani et al., 2014). Agents execute a series of methods at each time step: receive input from value layers, update network state, secrete latent TGFβ and IL-6, deposit collagen, migrate. Migration occurs randomly for all simulations, and cell proliferation and death are not simulated. One agent is allowed to occupy an individual grid space, and agent migration is confined to the borders of the simulation space. This series of methods is repeated for 1,008 time steps (6 weeks).

**Figure 2:**
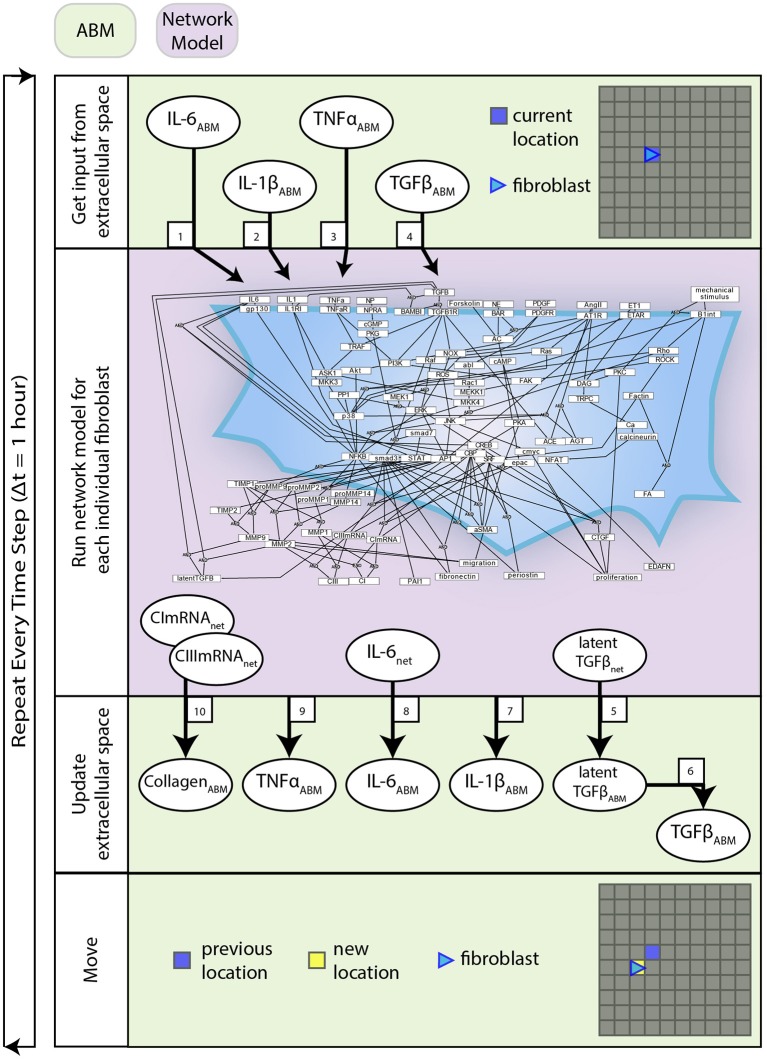
Coupled model process diagram. A detailed process diagram illustrates the methods and order in which they occur at each time step (1 h), and components of the ABM and network model that interact. Boxed numbers refer to the equation number which describes that process.

Interactions between the network model and ABM are described by Equations (1–10). These equations are used to define the behavior at the interface of the two models and are distinct from the equations that define the network model alone. The network model operates using normalized values between 0 and 1, whereas the ABM stores values in terms of physical concentrations. This set of equations act as a translator between these two systems. Figure 2 describes how these equations interact with components of each model and the order in which these methods are executed.

#### Network Model Inputs

Equations (1–4) are used to translate the cytokine levels stored as concentrations in the value layers of the ABM into inputs for the network model. Input weights for the network model range from 0 to 1, representing receptor activation between 0 and 100%. These weights are determined using the quantitative dissociation constants (Table 1) for the inputs of interest (IL-6, IL-1β, TNFα, and TGFβ). The dissociation constant is the concentration of ligand at which approximately half of the free ligand is bound to receptor at equilibrium. Receptor activity is described by a Hill equation, where a concentration of ligand equal to the K_d_ is considered 50% activation of the input node. Values for each of these dissociation constants are listed in Table 1.

(1) $$\begin{array}{l} w_{IL\text{-}6}=\;\frac{\left[IL\text{-}6_{ABM}\right]}{[IL\text{-}6_{ABM}]+K_{d,IL\text{-}6}} \end{array}$$

(2) $$\begin{array}{l} w_{IL\text{-}1\beta }=\;\frac{\left[IL\text{-}1\beta_{ABM}\right]}{[IL\text{-}1\beta_{ABM}]+K_{d,IL\text{-}1\beta }} \end{array}$$

(3) $$\begin{array}{l} w_{TNF\alpha }=\;\frac{\left[TNF\alpha_{ABM}\right]}{[TNF\alpha_{ABM}]+K_{d,TNF\alpha }} \end{array}$$

(4) $$\begin{array}{l} w_{TGF\beta }=\;\frac{\left[TGF\beta_{ABM}\right]}{[TGF\beta_{ABM}]+K_{d,TGF\beta }} \end{array}$$

**Table 1:** Dissociation constants.

**Parameter**	**Description**	**Equation #**	**Value**	**Unit**	**Value**	**Unit**	**Citation**
K_d,IL−6_	Dissociation constant for IL-6	1	22	nM	462,000	*pg*/*mL*	Baran et al., 2018
K_d,IL−1β_	Dissociation constant for IL-1β	2	500	pM	8,750	*pg*/*mL*	Dower et al., 1985; Issafras et al., 2014
K_d,TNFα_	Dissociation constant for TNFα	3	19	pM	323	*pg*/*mL*	Grell et al., 1998; Fallahi-Sichani et al., 2010
K_d,TGFβ_	Dissociation constant for TGFβ	4	28	pM	700	*pg*/*mL*	Wakefield et al., 1987

#### Active TGFβ and Latent TGFβ

Equation (5) describes the production of latent TGFβ from sources other than fibroblasts (k_gen_), secretion of latent TGFβ from fibroblasts (k_sec_) based on the network activity of latent TGFβ (latentTGFβ_net_), degradation of latent TGFβ (k_deg_), and activation of latent TGFβ (k_act_) based on the concentration of latent TGFβ in the ABM (latentTGFβ_ABM_). The generation rate (k_gen_) describes the production of latent TGFβ from sources that are not currently represented in this model (e.g., macrophages, neutrophils, etc.) and is used to maintain the gradient setup as described below under Initial Conditions. The secretion rate (k_sec_) describes the maximum physiological secretion of latent TGFβ from fibroblasts under stimulated conditions and this rate is scaled based on the network activity level (0–1) of latent TGFβ for each fibroblast. The degradation rate is a first-order rate based on the stability of latent TGFβ *in vitro*, and the activation rate describes the proportion of latent TGFβ that is converted to active TGFβ. Based on literature review, we chose a value for k_act,latentTGFb_ that maintains active TGFβ at 4–5% of total TGFβ, which is consistent with values measured in both *in vitro* and *in vivo* studies (Maeda et al., 2002; Hawinkels et al., 2007). In Equation (6), we use a rapid equilibrium assumption for active TGFβ concentration because the degradation rate of active TGFβ is on the order of minutes, much faster than our model time step of 1 h. Thus, we assume that the kinetics of active TGFβ are rate limited by the kinetics of latent TGFβ and come to a rapid quasi-equilibrium based on current latent TGFβ concentrations. Parameter values for Equations (5) and (6) can be found in Table 2.

(5) $$\begin{array}{l} \frac{{\partial latentTGF\beta }_{ABM}}{\partial t}=\;{k_{gen,\;latentTGF\beta }+k}_{\text{sec},latentTGF\beta }\\ \;*latentTGF\beta_{net}\\ \;-\;k_{\text{deg},\;latentTGF\beta }*latentTGF\beta_{ABM}\\ \;-k_{act,\;latentTGF\beta }*latentTGF\beta_{ABM} \end{array}$$

(6) $$\begin{array}{lll} TGF\beta_{ABM}\left(t\right) & = & k_{act,\;latentTGF\beta }*latentTGF\beta_{ABM}\left(t\right) \end{array}$$

**Table 2:** Parameters for active and latent TGFβ kinetics.

**Parameter**	**Description**	**Equation #**	**Value**	**Unit**	**Citation**
k_gen,latentTGFβ_	Generation rate of latent TGFβ required to create gradient	5	530,000	*pg*/mL*hr	Mass balance constraint
k_sec,latentTGFβ_	Latent TGFβ secreted by fibroblasts	5	23,700	*pg*/mL*hr	Wakefield et al., 1987; Campbell and Katwa, 1997; Campaner et al., 2006; Cartledge et al., 2015; Bolívar et al., 2017
k_deg,latentTGFβ_	First-order degradation rate for latent TGFβ	5	0.0096	*/hr*	Rollins et al., 1989
k_act,latentTGFβ_	Activation rate of latent TGFβ to active TGFβ	5 & 6	0.045		Maeda et al., 2002; Hawinkels et al., 2007

#### Inflammatory Cytokines

Equations (7–9) describe the production and degradation of IL-1β, IL-6, and TNFα. IL-1β and TNFα are not secreted by the current fibroblast network model, so these equations simply consist of a generation rate and first-order degradation rate. The generation rates are selected to maintain prescribed cytokine gradients as described below under Initial Conditions. The equation for IL-6 kinetics simply has the addition of a secretion rate that represents the maximum physiological secretion of IL-6 from fibroblasts under stimulated conditions and is scaled based on the network activity level (0–1) of IL-6 for each fibroblast. Parameter values for Equations (7–9) can be found in Table 3.

(7) $$\begin{array}{lll} \frac{{\partial IL-\text{1}\beta }_{ABM}}{\partial t} & = & \;k_{gen,\;IL\text{-}\text{1}\beta }-k_{deg,\;IL\text{-}\text{1}\beta }*{IL-\text{1}\beta }_{ABM} \end{array}$$

(8) $$\begin{array}{l} \frac{{\partial IL-\text{6}}_{ABM}}{\partial t}=\;k_{gen,\;IL\text{-}\text{6}}+\;k_{\sec,\;IL\text{6}}*{IL-\text{6}}_{net}-\\ \;k_{deg,\;IL6}*{IL-6}_{ABM} \end{array}$$

(9) $$\begin{array}{lll} \frac{{\partial TNF\alpha }_{ABM}}{\partial t} & = & \;k_{gen,\;TNF\alpha }-k_{deg,\;TNF\alpha }*TNF\alpha_{ABM} \end{array}$$

**Table 3:** Parameters for inflammatory cytokine kinetics.

**Parameter**	**Description**	**Equation #**	**Value**	**Unit**	**Citation**
k_gen,IL−1β_	Generation rate of IL-1β required to create gradient	7	4,847	*pg*/mL*hr	Mass balance constraint
k_deg,IL−1β_	First-order degradation rate for latent IL-1β	7	0.277	/*hr*	Hazuda et al., 1988; Friedman and Siewe, 2018
k_gen,IL−6_	Generation rate of IL-6 required to create gradient	8	256,000	*pg*/mL*hr	Mass balance constraint
k_sec,IL−6_	IL-6 secreted by fibroblasts	8	79,360	*pg*/mL*hr	Ancey et al., 2002; Fredj et al., 2005; Turner et al., 2007, 2009
k_deg,IL−6_	First-order degradation rate for IL-6	8	0.277	/*hr*	Gerhartz et al., 1994
k_gen,TNFα_	Generation rate of TNFα required to create gradient	9	895.4	*pg*/mL*hr	Mass balance constraint
k_deg,TNFα_	First-order degradation rate for TNFα	9	1.386	/*hr*	Zahn and Greischel, 1989

#### Collagen

Equation (10) describes the deposition and degradation of collagen in the ABM based on the collagen I and III mRNA nodes in the network model. Deposition of collagen occurs only where a fibroblast is present and is based on the value of the collagen I and III mRNA nodes in the network model for each fibroblast. Degradation is modeled as a first-order process based on the current collagen concentration in the ABM and thus occurs at every grid location, regardless of the presence of a fibroblast. This assumes evenly distributed MMP activity since we are not explicitly representing MMP production in this model.

These two parameters (Table 4) were fit based on previously published data in a rat model of MI (Fomovsky and Holmes, 2010). Baseline collagen area fraction was considered to be 4%, based on typical measurements from a healthy rat prior to an infarction (MacKenna et al., 1994). Collagen I and III mRNA activity levels from the network model run at a baseline condition for 6 weeks (0.25 for all input nodes) were used to fit the baseline experimental data. This was done by analytically solving equation 10 for the ratio of k_dep_/k_deg_ that produces a steady state collagen area fraction of 4%. Then, the network model was run for 6 weeks to simulate a stimulated condition (0.5 input for TGFβ, IL-1β, IL-6, and TNFα network nodes) and used to fit the infarct experimental data. This was accomplished by performing a parameter sweep of values for k_dep_ while constraining k_deg_ to satisfy the ratio determined previously and minimizing the sum of squared error (SSE) between the model fit and infarct experimental data.

(10) $$\begin{array}{l} \frac{{\partial Collagen}_{ABM}}{\partial t}=\;k_{dep,\;Collagen}\left(ColIRNA_{net}+ColIIIRNA_{net}\right)\\ \;-k_{deg,\;Collagen}*Collagen_{ABM} \end{array}$$

**Table 4:** Parameters for collagen deposition and degradation.

**Parameter**	**Description**	**Equation #**	**Value**	**Unit**	**Citation**
k_conv,Collagen_	Coefficient of collagen deposition	10	0.0056	Area fraction	Fit to exp. data
k_deg,Collagen_	Coefficient of collagen degradation	10	0.0035	Area fraction	Fit to exp. data

### Initial Conditions

**Figure 3:**
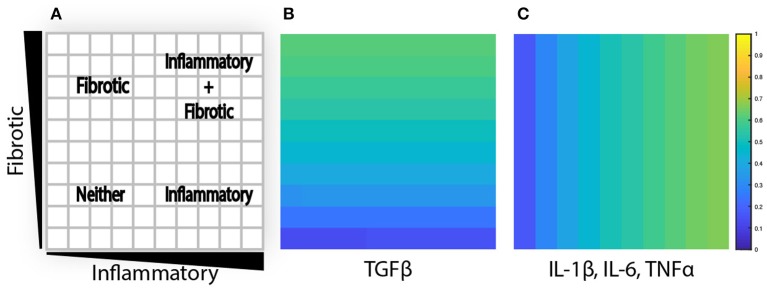
Agent-based model is initialized with cytokine gradients. **(A)** Four phenotypic regions are created by a combination of fibrotic and inflammatory cues. **(B)** TGFβ is initialized with an increasing gradient from bottom to top. **(C)** IL-1β, IL-6, and TNFα are initialized with an increasing gradient from left to right.

To evaluate the effects of spatial gradients in fibrotic and inflammatory cues, the value layers are initialized with a gradient of TGFβ increasing from bottom to top, and a gradient of inflammatory cytokines (IL-1β, IL-6, and TNFα) increasing from left to right. Thus, each individual grid space contains a unique combination of fibrotic and inflammatory cues. Cytokine gradients were specified by scaling the generation rate (k_gen_) along the *x* or *y* axis to result in concentrations ranging up to twice the dissociation constant for that particular cytokine or growth factor, corresponding to receptor activation rates between ~16 and 67% (Figures 3A–C). The purpose is 2-fold: to explore a dynamic range of inputs to the network model and to create an environment where fibroblasts migrate through spatially varying environmental cues.

### Parameter Estimation and Fitting

A total of 17 parameters are defined in this set of 10 equations. Of these parameters, 11 are based on literature and 6 are estimated, or fit to experimental data, as noted in Tables 1–4. The dissociation constants, degradation rates, and production rates from fibroblasts are based on literature. The generation rates are estimated based on a mass balance constraint in order to create the specified initial gradient, which are ultimately based on the dissociation constants found in literature. Coefficients for collagen deposition and degradation are fit based on experimental data.

### Sensitivity Analysis

A sensitivity analysis of all 17 model parameters was conducted. Each parameter was decreased individually by an order of magnitude (0.1×) and compared to the results of a model run with all parameters at baseline values. A sensitivity coefficient was calculated using Equation (11), where *y*_o_ and *y*_i_ are the measured state variable when parameters are at baseline or perturbed, respectively, and *p*_o_ and *p*_i_ are the values of the baseline parameter and perturbed parameter.

(11) $$\begin{array}{l} S=\;\frac{y_{i}-y_{o}}{p_{i}-p_{o}}*\frac{p_{o}}{y_{o}} \end{array}$$

The state variables measured in the sensitivity analysis are the total collagen content, global semivariance (r_xy_), and semivariance in either the x (r_x_) or y (r_y_) dimension. Collagen content is measured by summing collagen area fraction across all individual grid spaces. Global semivariance is (*r*_xy_) defined as:

(12) $$\begin{array}{lll} r_{xy} & = & \frac{1}{2s_{0}}\underset{i}{\sum }\underset{j}{\sum }W_{ij}{\left(x_{i}-x_{j}\right)}^{2}\\ s_{0} & = & \underset{i}{\sum }\underset{j}{\sum }W_{ij} \end{array}$$

where *x*_i_ is an observed data point, *x*_j_ is an adjacent observation, *W*_ij_ is a matrix of spatial weights, and s_0_ is the sum of all *W*_ij_. If two data points are immediate neighbors, *W*_ij_ is assigned as 1, otherwise *W*_ij_ is set to 0 (Lee et al., 2019). Semivariance in the *x* dimension is calculated by assigning 1 to *W*_ij_ for adjacent observations in the *x* direction and 0 otherwise, and vice versa for the *y* dimension.

### Model Implementation

This model was implemented using Repast Simphony 2.6 with a java engine to connect to MATLAB R2018b, which was used to run the network model and perform all data analysis. All simulations here were performed on a single CPU (Intel^®^ Xeon^®^ E5-2640 v4@2.4GHz). The approximate runtime to simulate 100 fibroblasts for a period of 6 weeks is 1 h and 17 min.

### Cardiac Fibroblast *in vitro* Experiments

Primary human ventricular cardiac fibroblasts were purchased from PromoCell (PromoCell C-12375; PromoCell GmbH, Germany). Cells were cultured in DMEM containing 10% FBS and 1% Pen/Strep, and were kept in an incubator maintained at 5% CO_2_. Cells were plated in a 96-well plate at 5,000 cells/well and then grown in 10% FBS for 24 h, serum starved for 24 h, and then treated with the following conditions for 96 h: 0%FBS control media, 0%FBS media with 20 ng/mL TGFβ1 (Cell Signaling Technology, 8915LC), and 0% FBS media with 10 ng/mL human IL1β (Cell Signaling Technology, 8900SC). Cells were then fixed in 4% PFA in PBS for 30 min, permeabilized and blocked for 1 h in a solution containing 3% BSA and 0.2% Triton, and then stained overnight at 4°C with a 1:500 primary Anti-Collagen I antibody (Abcam, ab34710). After an overnight incubation, cells were washed 3 × in PBS and stained with 1:5,000 Dapi, 1:1,000 Phalloidin CruzFluor 647 stain (Santa Cruz Biotechnology, sc-363797), 1:250 α-Smooth Muscle Actin preconjugated antibody (Sigma-Aldrich, C6198), and 1:1000 Goat-anti-Rabbit (secondary for Anti-Collagen I) (ThermoFisher Scientific, A-11034).

### Microscopy and Image Analysis

96-well-plates we imaged using the Operetta CLS High-Content Analysis System with confocal view, and Dapi, Alexa 488, TRITC, and Alexa 647 imaging channels (Perkin Elmer). Three wells for each condition were imaged and quantified. To identify individual cells, an automated image analysis pipeline was employed in CellProfiler (Carpenter et al., 2006). Fibroblast nuclei were identified by DAPI signal, and fibroblast boundaries corresponding to each nuclei were segmented based on collagen and phalloidin (actin) signals using the “propagate” algorithm. αSMA, pro-collagen I, and phalloidin signals were integrated within each cell's boundary to determine fluorescence per cell. To reduce error from edge effects, only cells in the center tile of each well were measured. The median fluorescence for all cells in a given well was reported (*n* = 3 replicate wells per treatment group, 250–450 cells per well were imaged). Significance between groups was determined by one-way ANOVA with Tukey HSD *post-hoc* test, *p* ≤ 0.05 considered significant. Data are provided in Supplemental Table 1.

## Results

### Coupled Model Can Reproduce Predictions Made by Network Model Alone

**Figure 4:**
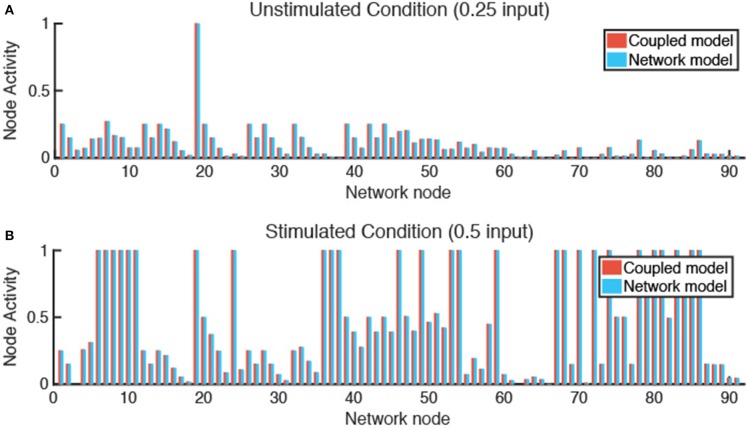
Verification tests confirm that coupled model and network model produce equivalent fibroblast network states. **(A)** In an unstimulated condition (0.25 input for all nodes), and a **(B)** stimulated condition (0.5 input for TGFβ, TNFα, IL-1β, and IL-6 nodes), the network state of a fibroblast using the coupled model or network model alone are comparable with a SSE of 3.865e-7 **(A)** and 1.168e-6 **(B)**.

Verification tests were performed to evaluate whether coupling of LDE and ABM models affected results obtained from each model individually. This was accomplished by seeding one fibroblast in each grid space with no migration and simulating either an unstimulated condition (0.25 input for all nodes) or stimulated condition (0.5 input for TGFβ, IL-1β, IL-6, and TNFα). We compared the activity level of all 91 nodes of the fibroblast network state at steady state for each condition and calculated the sum of squared error (SSE) between the network-only and coupled models. The SSE for the unstimulated condition is 3.865e-7 (Figure 4A) and 1.168e-6 for the stimulated condition (Figure 4B). We next examined the SSE between the network states of the coupled model and network model alone for all 100 combinations of cytokine inputs from Figure 3, resulting in SSEs in the range of [2.07e-8–8.88e-5]. Thus, the network-only and coupled models produce equivalent network states for non-migrating fibroblasts when cytokine inputs are maintained at a constant level for individual cells.

### Coupled Model Predicts That Inflammatory Cytokines Antagonize TGFβ-Induced Collagen Accumulation

**Figure 5:**
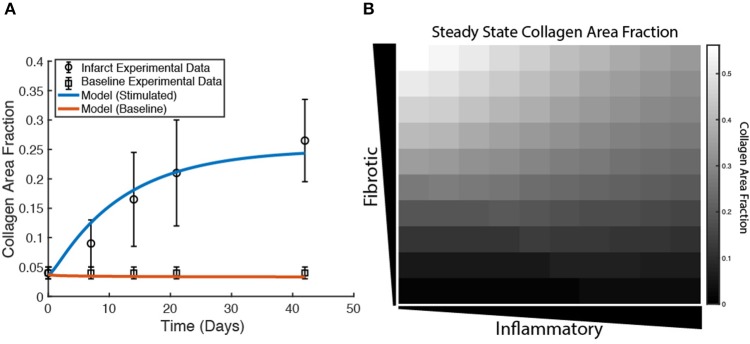
Coupled model predicts collagen profile over a range of physiological conditions. **(A)** Collagen area fraction for an unstimulated condition (0.25 input for all nodes) is compared to baseline collagen area fraction (4%) in a healthy rat. Model predictions for a stimulated condition (0.5 input for TGFβ, TNFα, IL-1β, and IL-6 nodes) are compared to results from a rat model of myocardial infarction up to 6 weeks post-MI. Error bars = SEM. **(B)** Collagen area fraction predictions at 6 weeks from a model simulation with gradient initial conditions and a fibroblast in each grid space (*n* = 100).

Model parameters for collagen deposition and degradation were fit to match experimental data obtained previously from a rat model of MI (Fomovsky and Holmes, 2010). For these simulations, a single fibroblast was placed in every grid space and not allowed to migrate. Model simulations were initialized to match the measured rise in collagen area fraction in healing infarcts when fibrotic and inflammatory inputs to the coupled model (TGFβ, IL-1β, IL-6, and TNFα) were maintained at an elevated level of 0.5, and to match the normal myocardial collagen area fraction when the same inputs were maintained at their baseline values of 0.25 (Figure 5A). Expanding to a broader range of cytokine combinations (100 combinations of fibrotic vs. inflammatory cytokines), the coupled model predicted biologically plausible variations in steady-state collagen content. As shown in Figure 5B, collagen content is highest in areas with high TGFβ input and low inflammatory input, and is reduced as inflammatory input increases for the same magnitude of TGFβ input.

### Crosstalk Between TGFβ and Inflammatory Extracellular Cues Produces Complex Signaling Behaviors

**Figure 6:**
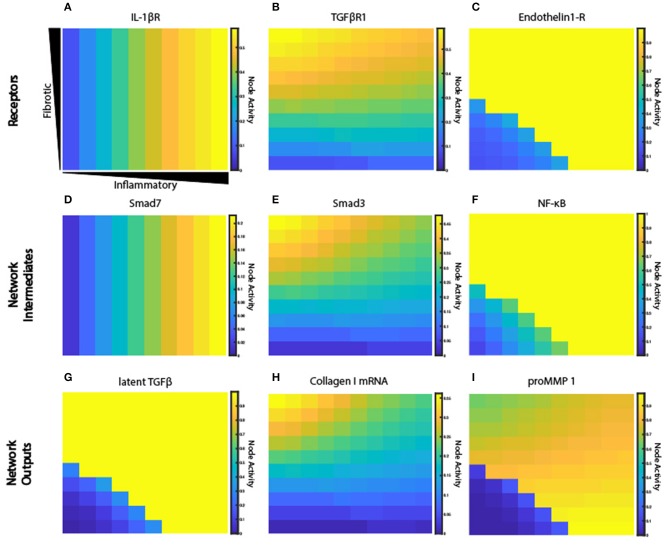
Signaling network exhibits a range of activation patterns in response to extracellular cues. Node activity level of individual network nodes for each fibroblast at steady state (6 weeks). Model simulation with gradient initial conditions and a fibroblast in each grid space (*n* = 100). Heat maps show network states for input receptors **(A–C)**, intermediate network nodes **(D–F)**, and network outputs **(G–I)**.

The fibroblast network model exhibits complex behaviors due to its integration of 10 interdependent signaling pathways. Figure 6 illustrates representative nodes in the fibroblast network and their activity level under conditions of constant cytokine inputs for many combinations of inflammatory and fibrotic inputs, as described previously. Network receptors display a range of activation patterns based on their extracellular cues (Figures 6A–C). IL-1β receptor activation closely follows the gradient created by the initial conditions. In contrast, TGFβR1 and endothelin-1 (ET-1) are influenced by autocrine feedback loops. Inflammatory cytokines cause inhibition of TGFβR1 that increases along the x-axis as the concentration of inflammatory cytokines increase. TGFβR1 activation is also influenced by the rate of latent TGFβ activation, which occurs in the ABM value layers. Fibroblasts secrete latent TGFβ, which is then activated to active TGFβ. But as noted in Figure 6G there is minimal latent TGFβ produced in environments of low TGFβ and inflammatory input. As a result, there is decreased TGFβR1 activation in this quadrant. In contrast to the gradual applied input gradients, ET-1 receptor displays switch-like activation, due to autocrine feedback of activator protein 1 (AP1) downstream of both TGFβ and IL-1β.

Some nodes downstream of each of these inputs display similar activation patterns (Figures 6D–F), while others integrate multiple unique upstream inputs. Smad7, which is immediately downstream of STAT and IL-1β receptor, displays an activity pattern similar to the IL-1β receptor. Smad3 is downstream of TGFβR1, and it regulates many network outputs including collagen mRNA, fibronectin, periostin, and αSMA. NF-κB activity is regulated by many inputs, including IL-1βR, ERK, p38, and AKT. But ERK and p38 (which are immediately downstream of the ET-1 receptor) dominate the response of NF-κB, so it displays an activation pattern most similar to ET-1 receptor. NF-κB contributes to the expression of MMPs, fibronectin, and provides feedback to IL-6 input.

Network outputs represent the integration of many upstream inputs (Figures 6G–I). Latent TGFβ expression is primarily influenced by AP1 transcriptional activity, which itself is regulated by ERK and JNK. The model predicts that IL-1β antagonizes TGFβ-induced collagen I mRNA and αSMA mRNA, which is validated by experimental studies in lung and dermal fibroblasts (Mia et al., 2014). Expression of collagen I mRNA is predicted to be a product of input from Smad3, SRF (serum response factor), and CBP (CREB binding protein). proMMP 1 expression is a prime example of integration of multiple upstream inputs that each exhibit distinct activation patterns including AP1, Smad3, and NF-κB. Visualization of how this combination of transcription factors regulates proMMP 1 expression is shown in Supplementary Figure 1. In summary, the coupled model provides a platform to investigate how combinations of dynamic inputs affect downstream intermediate network nodes and network outputs.

### Key Parameters Affect Spatial Gradient of Collagen Deposition

**Figure 7:**
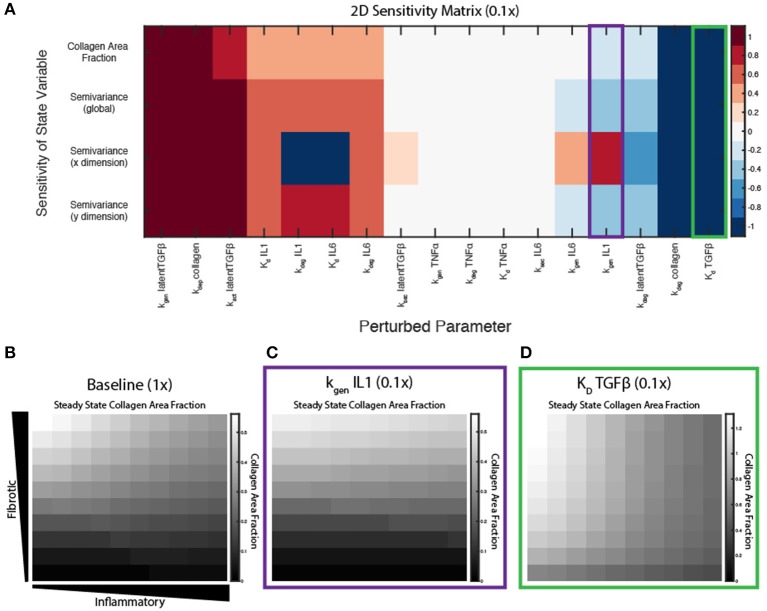
Key parameters affect spatial gradient of collagen deposition. **(A)** Sensitivity coefficients calculated based on Equation (11) with individual parameter perturbations of 0.1×. State variable outputs include total collagen area fraction, global semivariance, semivariance in the *x* direction, and semivariance in the *y* direction. Collagen area fraction heat maps at 6 weeks with **(B)** all parameters at baseline, **(C)** k_gen,IL−1β_ parameter multiplied by 0.1, and **(D)** K_d,TGFβ_ parameter multiplied by 0.1.

A sensitivity analysis was conducted to determine the relative influence of decreasing the values of parameters associated with ABM-network coupling on overall collagen content (area fraction) and collagen heterogeneity (semivariance, either globally, or in the *x* or *y* dimension). Parameters were individually decreased by an order of magnitude (0.1×), and normalized sensitivity coefficients were computed, in which positive coefficients indicate positive correlation of the parameter with the output measured (see Equation 11). Parameters were ranked by their positive influence on collagen area fraction (Figure 7A). Parameters related to TGFβ production, activation, and degradation are the most influential in determining collagen content, because TGFβ input is important in altering downstream collagen I and III mRNA activity in the fibroblast signaling network. As expected, the coupled model is also highly sensitive to the two parameters in Equation (10) that govern deposition and degradation in the collagen layer. In terms of inflammatory inputs, this analysis reveals that IL-1β and IL-6 are more influential in determining the collagen profile than TNFα input. This is likely because IL-6 has a downstream effect on Smad3, which promotes collagen mRNA activity, and IL-1β upregulates NF-κB, which has a positive feedback on IL-6. TNFα has a smaller effect on NF-κB signaling and no direct connection to Smad3 signaling. It is also noted that the two parameters related to secretion of latent TGFβ and IL-6 (k_sec,latentTGFβ_ and k_sec,IL−6_) from the fibroblast have little effect on the overall collagen profile, yet TGFβ and IL-6 inputs themselves seem to be very influential. This is likely because the production rates from fibroblasts are not high enough to significantly impact the gradients that are created in the initial conditions.

Interestingly, some parameters may have different effects on overall collagen content and collagen spatial heterogeneity. Decreasing parameters such as the K_d_ of TGFβ for its receptor, increase both collagen content and collagen heterogeneity (Figures 7A,D), compared to a collagen profile at 6 weeks when all parameters are at baseline values (Figure 7B). Decreasing parameters associated with synthesis of latent TGFβ or collagen, or degradation of IL-6, cause a decrease in both collagen content and heterogeneity in both dimensions. In contrast, decreasing the IL-1 generation term (k_gen,IL−1_) has little effect on the total collagen content, but has opposite effects on collagen heterogeneity in the *x* and *y* dimension, as measured by semivariance in either direction (Figure 7C).

### Single Cell Dynamics in Response to a Changing Extracellular Environment

**Figure 8:**
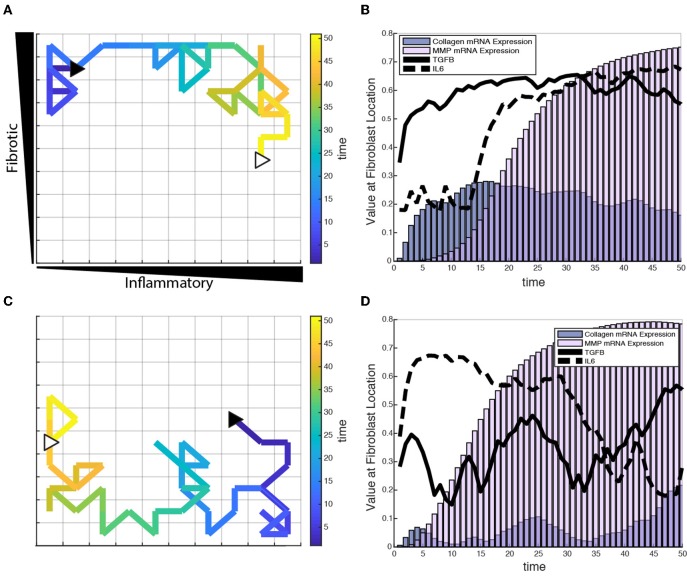
Individual fibroblasts respond dynamically to extracellular environment. **(A,C)** Fibroblast migration path for a single fibroblast over a period of 50 h. Fibroblast starting location indicated by filled black triangle and end location indicated by open white triangle. **(B,D)** Corresponding TGFβ and IL-6 inputs for the fibroblasts tracked in **(A,C)**, and their respective collagen and MMP mRNA activity over the time course of 50 h.

To test the role of fibroblast migration on collagen remodeling, fibroblasts were seeded sparsely in the coupled model and allowed to migrate stochastically at a rate of one grid space per hour. Fibroblasts were seeded at moderate density (20 fibroblasts) within the fibrotic vs. inflammatory cytokine grid and responses simulated for 6 weeks. Fibroblasts experience changes in their extracellular environment as they migrate, which causes their intracellular signaling network state and rate of collagen deposition to change accordingly. Figure 8 illustrates single cell migration trajectories (panels A and C), local cytokine inputs, and gene expression (panels B and D) for two representative fibroblasts migrating within the cytokine gradient environment. The fibroblast shown in panels A and B remains in areas with high to moderate TGFβ levels and with increasing levels of inflammatory cytokines. Correspondingly, this fibroblast exhibited relatively high levels of collagen mRNA expression that mirrored the level of TGFβ input. As this fibroblast migrated to regions of increasing IL-6, there was a delayed but then rapid increase in MMP mRNA expression, consistent with switch-like responses seen in Figure 6I.

Figures 8C,D track a separate fibroblast that remains in areas of low to moderate TGFβ levels, but migrates from a region of high to low inflammatory inputs. This simulation shows similarly that collagen mRNA expression closely follows TGFβ inputs. Interestingly, it also demonstrates that exposure to high IL-6 levels triggers a rapid and sustained increase in MMP mRNA expression, which persists well after the cell migrates to a region with lower levels of inflammatory cytokines. Thus, some network nodes respond with close coordination to particular cytokine inputs, whereas other nodes may be activated in a switch-like manner consistent with the activation patterns seen in Figure 6. Supplementary Videos 1, 2 offer a visual of how the entire network state changes over time for the individual fibroblasts presented in Figure 8.

### Fibroblast Migration Speed and Density Affect Collagen Spatial Heterogeneity

**Figure 9:**
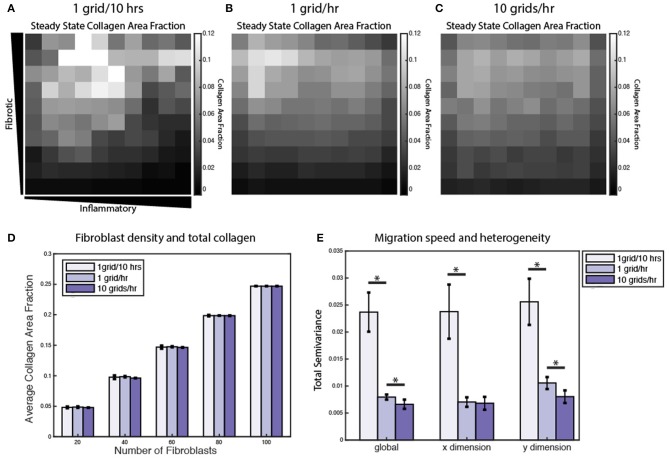
Fibroblast migration speed and density affect spatial heterogeneity of collagen. Collagen area fraction heat map at 6 weeks for simulations with 20 randomly migrating fibroblasts with a migration speed of **(A)** 1 grid/10 h, **(B)** 1 grid/h, and **(C)** 10 grids/h. **(D)** Average collagen area fraction at 6 weeks for each migration speed and simulations with 20, 40, 60, 80, or 100 fibroblasts. Mean reported for 5 runs. Error bars = standard deviation. **(E)** Semivariance calculated globally, in the *x* direction, and *y* direction for each migration speed. Mean reported for 10 runs. Error bars = standard deviation. ^*^p < 0.05.

Under normal conditions, fibroblasts migrate at a speed of ~10 μm/h, but this can vary significantly in the presence of growth factors and cytokines (Ware et al., 1998; Pérez-Rodríguez et al., 2018). As noted above, individual grid spaces are 10 × 10 μm, so the baseline migration speed was set at 1 grid space per hour. To test the impact of altered migration speed, migration speeds were set to default values (1 grid/h), decreased (1 grid space/10 h) or increased (10 grid spaces/h). Slower migration speed resulted in greater heterogeneity in the collagen profile, while faster migration speed resulted in a more homogenous collagen profile (Figures 9A–C). In contrast, overall collagen content was linearly dependent on fibroblast density, but not migration speed or initial cell location (*n* = 10 simulations per condition) (Figure 9D). As with the sensitivity analyses, heterogeneity in collagen was quantified by global, x-direction, or y-direction semivariance (see Equation 12). Consistent with qualitative observations from Figures 9A–C, decreasing the migration speed enhanced both the average magnitude and run-to-run variance in collagen heterogeneity (Figure 9E). Faster migration decreased collagen heterogeneity globally and in the *y* dimension, but not in the *x* dimension. The coupled model predicts that fibroblast migration can have a substantial impact on the spatial heterogeneity and stochasticity of collagen deposition.

### A Subset of Coupled Model Predictions Was Validated by Comparison to *in vitro* Experiments

**Figure 10:**
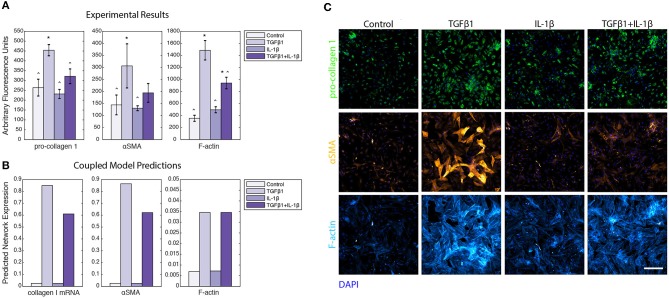
Coupled model predictions were compared to independent *in vitro* experiments using human cardiac fibroblasts treated with TGFβ1 and/or IL-1β. **(A)** Pro**-**collagen 1, αSMA, and F-actin expression from *in vitro* experiments with human cardiac fibroblasts were quantified by image analysis to measure the median fluorescence for all individual cells in each well (*n* = 3). Treatment conditions included control, TGFβ1 (20 ng/mL), IL-1β (10 ng/mL), and TGFβ1 (20 ng/mL) + IL-1β (10 ng/mL). Error bars = standard deviation. ^*^p < 0.05 with reference to control condition. ^∧^*p* < 0.05 with reference to TGFβ1 condition. **(B)** Coupled model predicts network expression of collagen I mRNA, αSMA, and F-actin when simulating the addition of TGFβ1 (20 ng/mL), IL-1β (10 ng/mL), and TGFβ1 (20 ng/mL) + IL-1β (10 ng/mL), compared to a simulation with all parameters at baseline. **(C)** Representative images of human cardiac fibroblast expression of pro-collagen 1 (green), αSMA (orange), and F-actin (purple) when treated with TGFβ1 (20 ng/mL), IL-1β (10 ng/mL), and TGFβ1 (20 ng/mL) +IL-1β (10 ng/mL), compared to control. Nuclei are stained with DAPI (blue). Scale bar = 500 microns.

In order to perform experimental validation of our coupled model predictions, we ran a series of simulations wherein input cytokine (IL-1β) and growth factor (TGFβ1) levels were varied from baseline to simulate those tested with *in vitro* experiments using primary human cardiac fibroblasts (HCFs). HCFs were treated with either TGFβ1 (20 ng/mL), IL-1β (10 ng/mL), or TGFβ1 (20 ng/mL) + IL-1β (10 ng/mL), and compared to a control condition in media without FBS (since this is also a source of TGFβ1). Pro-collagen I, αSMA, and F-actin expression were quantified using immunocytochemistry and image processing to quantify the median fluorescence in each of these experimental conditions (Figure 10A). Treatment with TGFβ1 significantly increased expression of pro-collagen I, αSMA, and F-actin compared to the control condition, while IL-1β treatment alone had no significant effect on pro-collagen I, αSMA, or F-actin expression when compared to the control condition. The combination of TGFβ1 and IL-1β treatment decreased expression of pro-collagen I, αSMA, and F-actin when compared to TGFβ1-only treatment, and was statistically significant in the cases of pro-collagen I and F-actin. Representative images of pro-collagen 1 (green), αSMA (orange), and F-actin (blue) expression in HCFs for each of these treatment conditions indicate the trends described above (Figure 10C). These experimental measurements were compared to *in silico* predictions that simulated the addition of these factors at the same concentrations tested experimentally: TGFβ1 (20 ng/mL), IL-1β (10 ng/mL), or TGFβ1 (20 ng/mL) + IL-1β (10 ng/mL). As with the experimental results, predictions were compared to a control simulation in which all parameters were set to baseline levels (Figure 10B). Most model predictions qualitatively agreed with the trends observed with *in vitro* experiments. For example, network expression of collagen I mRNA, αSMA, and F-actin were increased relative to the control simulation in response to TGFβ1 stimulation. Similar to observed experimental results, IL-1β treatment alone had no effect on collagen I mRNA, αSMA, or F-actin network expression compared to the control simulation, but the combination of TGFβ1 and IL-1β treatment was predicted to decrease the expression of collagen I mRNA and αSMA when compared to TGFβ1 only treatment. Unlike experimental results, however, simulating this combined treatment predicted no change in F-actin network expression when compared to simulating TGFβ1 only treatment. These model predictions were further validated by published experimental studies wherein IL-1β attenuated TGFβ1-induced collagen I synthesis and αSMA expression of lung and dermal fibroblasts (Mia et al., 2014).

## Discussion

### A Novel Hybrid Multiscale Model of Tissue Fibrosis

Here we present a novel hybrid multiscale model of tissue fibrosis that couples a LDE model of cardiac fibroblast intracellular signaling with an ABM of multi-cellular tissue remodeling. Prescribed gradients of inflammatory cues (IL-1β, IL-6, and TNFα) and fibrotic cues (TGFβ) stimulate migrating fibroblasts to respond dynamically to their locally varying extracellular environment. Under conditions with no fibroblast migration and constant cytokine input levels, the coupled model was verified to exhibit consistent network states predicted by the network model alone (Figure 4). In contrast, the addition of fibroblast migration across a gradient of cytokine inputs demonstrated that fibroblasts respond dynamically to both their local cytokine environment and their previous history of cytokine exposure. Spatial heterogeneity of collagen was dependent on the speed of fibroblast migration and key parameters (e.g., Il-1β synthesis rate) identified in a sensitivity analysis as having distinct effects on semivariance in the *x* or *y* dimension. Additionally, several parameters were identified to be influential in contributing to the overall amount of collagen deposition, including cell density and model parameters related to TGFβ production and activation.

The effects of pro-fibrotic stimuli (TGFβ) on increasing collagen expression and other myofibroblast markers is well-established (Reed et al., 1994; Chen et al., 2000; Thannickal et al., 2003). However, the crosstalk of IL-1β with other fibrotic signaling pathways is not as well-described. This coupled model provides a framework for investigating the effects of combined inflammatory and pro-fibrotic cues on spatial fibroblast activity and ECM composition. A subset of coupled model predictions was validated by comparison to experiments with human cardiac fibroblasts treated with combinations of TGFβ1 and IL-1β *in vitro*. The model accurately predicted TGFβ1-enhanced expression pro-collagen I, αSMA, and F-actin, as well as negative crosstalk on pro-collagen I and αSMA by IL-1β. However, the model did not predict the experimentally-observed attenuation of TGFβ1-enhanced F-actin expression by IL-1β, suggesting that additional cross-talk mechanisms may need to be explored in future experiments and model revisions.

Previous mathematical models of fibrosis have used deterministic, continuum methods to holistically represent the complex processes of fibrosis (Hao et al., 2015; Friedman and Hao, 2017). For example, Hao et al., describe a model of liver fibrosis using a system of 24 partial differential equations (PDEs) that represent many different cell types, cytokines and growth factors, and interactions between cells, and then use this model to interrogate different treatment options (Friedman and Hao, 2017). Other continuum-based models have focused more on specific mechanisms that contribute to the progression of fibrosis, such as macrophage activation and polarization (Jin et al., 2011; Wang et al., 2012). A model developed by Wang et al., for example, explores how the timing of monocyte recruitment and macrophage differentiation affects left ventricular remodeling following MI (Wang et al., 2012). However, an interesting study by Figueredo et al. suggested that stochastic differential equation approaches that assume continuous space and time could not capture the individual variability and spatial heterogeneity predicted by an agent-based modeling approach applied to the same biological case study, and that emergent behavior of the ABM contributed additional insight about the system (Figueredo et al., 2014).

An increasing number of hybrid models couple continuum with discrete approaches. These hybrid models typically couple ABMs, which use a discrete representation of 2D space or 3D volumes, with continuum based approaches that represent cytokine gradients and/or receptor-ligand kinetics (Warsinske et al., 2016, 2017; Virgilio et al., 2018). For example, Warsinske et al. simulated granuloma-associated fibrosis by incorporating a system of ODEs and PDEs that describe molecular level diffusion of chemokines (TGFβ and prostaglandin) and receptor ligand signaling, coupled with discrete cellular agents whose behaviors were defined by a set of rules that related receptor activation levels to cell proliferation, differentiation, chemotaxis, and secretion of ECM proteins. In these hybrid continuum-ABM models, outcomes at the tissue scale are the emergent product of actions of the individual agents governed by rules that are informed by molecular scale interactions simulated using continuum assumptions (Warsinske et al., 2017).

The hybrid multiscale model presented here represents the coupling of a large-scale intracellular network model, comprising 10 cytokine/neurohormonal inputs and 134 reactions, with an ABM that maps physiologically relevant *in vitro* concentrations of cytokines and ECM components to normalized network activity levels and vice versa. We believe that this represents the first coupling of a large-scale network model to make predictions about tissue-level changes in extracellular matrix composition in the setting of fibrosis. This coupled model and its use of concentration scaling between the logic-based model and physical units enables the quantitative prediction of fibroblast production of cytokines and growth factors and spatial gradients of cytokine concentrations, which was not previously possible with the network model alone. This coupled model framework will ultimately enable quantitative comparisons of model predictions to *in vivo* experimental data such as measurements of multiple cytokine concentrations over time, spatial profiles and gradients of ECM components, cell densities, and single cell mRNA expression.

### Impact of Spatially Varying Environmental Cues on Fibroblast Signaling

The response of a complex signaling network to multiple simultaneous cues is rarely intuitive, and we have demonstrated that individual nodes of the signaling network respond with distinct patterns of activation (Figure 6). Some receptors respond in sync with their input, such as IL-1βR, whose activity level mimics the gradient initial conditions of IL-1β input. Meanwhile other receptors, such as TGFβR1, display a more complex pattern of activation reflecting not only the gradient initial conditions but also feedback from latent TGFβ activation and inhibition by IL-1 activity. Intermediate nodes often display a similar pattern of activation to their immediate upstream receptors (e.g., Smad7 and IL-1βR, Smad3 and TGFβR1, NF-κB and Endothelin1-R), while network outputs integrate the effects of many upstream network nodes that represent a combination of stimulatory and inhibitory inputs. MMP1, for example, is upregulated by NF-κB and AP1 (activator protein 1) activity, and inhibited by Smad3 activity. Tracking the response of individual fibroblasts moving through varying levels of inflammatory and fibrotic inputs revealed a complex kinetic relationship between the locally sensed extracellular environment and network state of a migrating fibroblast (Figure 8 and Supplementary Videos 1, 2). For example, a fibroblast that experiences a high inflammatory context will upregulate its MMP activity, which remains elevated even if the fibroblast moves to an environment with low inflammatory and fibrotic inputs. The fibroblast's network state is highly dependent on the current extracellular environment in some cases (e.g., collagen mRNA expression in response to TGFβ input) but displays history-dependence of previous environments in other cases.

### Processes That Contribute to Spatial Heterogeneity of Collagen Deposition

The artificially prescribed cytokine gradient environment employed in these simulations (Figure 3) was not intended to represent a particular *in vivo* situation, but was used to evaluate the ability of the coupled signaling and multicellular model to predict the progression of fibrosis across a wide range of signaling contexts. Thus, changes in heterogeneity discussed here reflect the range of responses a population of fibroblasts would be expected to generate across those varied signaling contexts (Figure 5B). For example, we found fibroblast migration speed to be an important determinant of collagen heterogeneity in our simulations (Figure 9). Slower migration speed leads to pockets of high collagen deposition and overall higher heterogeneity. Faster migration produces a more uniform collagen distribution. In healing wounds where cytokine concentrations vary in both space and time, we expect that high migration speeds could similarly blur the effects of variable cytokine levels while slow migration speeds could accentuate them. In contrast, migration speed did not substantially affect average collagen accumulation across the entire simulated range of cytokine combinations. Rather, overall collagen accumulation was strongly dependent on fibroblast density. In addition to fibroblast density, model parameters related to TGFβ production, activation, and degradation are among the most important model parameters in determining total collagen content as well as the gradient of collagen deposition in either dimension (Figure 7), which agrees with the findings from similar models of fibrosis (Warsinske et al., 2016). Other parameters such as the degradation or synthesis of IL-1β had opposite effects on collagen heterogeneity in two dimensions. One advantage of coupling an ABM is that it produces stochastic predictions as a result of individual-based rule sets and a spatial context (Supplementary Figure 2). Repeated runs of the coupled model may help to capture individual variability of spatial fibrosis seen in animal models.

### Computational Requirements for Scaling Up

Simulations were performed on a single CPU (Intel^®^ Xeon^®^ E5-2640 v4 @2.4GHz). The runtime for 100 fibroblasts for a period of 6 weeks with access to 10 cores is 1 h and 17 min. This model is structured such that parallel computing can be implemented across multiple CPUs to reduce simulation runtime. The limit of computational efficiency is reached when the number of cores is equal to the number of fibroblasts in a simulation, allowing the network state of each fibroblast to be updated simultaneously at each time step. We anticipate that with access to 100 cores, this model with 100 fibroblasts for 6 weeks of simulated time would be computed in ~8 min. Simulation on a high-performance computing system with thousands of cores is expected to enable simulation of up to 100,000 fibroblasts (comparable to a myocardial infarct).

### Limitations and Sources of Error

The prescribed input cytokine gradients employed here were used to explore the dynamic range of the network model and create an environment where fibroblasts migrate through a rapidly changing extracellular environment. However, this environment is not representative of a specific physiological environment. Cytokine diffusion was not enabled in the current simulations, in order to maintain the prescribed cytokine input gradients over small spatial area (100 × 100 μm). Furthermore, the current model did not include proliferation and apoptosis, which have been simulated in previous work (Warsinske et al., 2016, 2017). Future applications will incorporate cell migration and proliferation rates that are driven by the dynamic network state of individual fibroblasts (Bailey et al., 2007, 2009). Additionally, this model focused specifically on the contributions of fibroblasts in the progression of fibrosis, through the coupling of a fibroblast signaling network, but future work will incorporate inflammatory cells that serve as local sources of many of the inflammatory cytokines that affect fibroblast signaling (Virgilio et al., 2018).

### State of the Multiscale Modeling Field and Contributions of This Multiscale Model

A primary focus of the computational modeling community is to develop methods for integrating biological data across spatial, temporal, and functional scales (Walpole et al., 2013). Continued advancements in the capabilities and availability of high-performance computing has allowed models to tackle more complex problems with greater resolution. Perturbations to fine-grained parameters, such as protein or gene expression data, can predict observable changes to coarse-grained parameters (e.g., cell distributions, tissue patterning) (Stern et al., 2012; Walpole et al., 2017; Warsinske et al., 2017). Furthermore, the use of multiscale models allows for *in silico* predictions for a wide range of parameter values in a high-throughput manner that would otherwise not be feasible with experimental assays, either due to time or cost constraints, or lack of the appropriate technology. For example, the multiscale model presented here allows for real time tracking of individual fibroblasts and continuous measurements of their network states, which would not be feasible *in vivo*. While models cannot fully replace experimental studies, they can offer insight into unexpected predictions that can then be experimentally tested or lead to new hypotheses entirely, as demonstrated by Martin et al., who predicted a new therapeutic approach as a result of their *in silico* experiments of muscle regeneration following injury (Martin et al., 2017).

In summary, we have contributed a hybrid multiscale model of tissue fibrosis by coupling models across spatial and temporal scales. This represents the coupling of a large-scale network model with an ABM to make predictions about fibroblast production of cytokines and growth factors and tissue-level changes in ECM composition. This coupled model makes predictions about fibroblast production of cytokines and growth factors in physical units, which was not possible previously with the logic-based model alone. Verification tests confirmed that the model coupling did not disrupt the behavior of the individual models, allowing for future model revisions or software implementations of individual modules. Application of this coupled model in the context of post-MI wound healing will allow for further investigation and validation of cytokine concentrations, collagen content and heterogeneity, and cell behaviors with both fine spatial and temporal resolution. Experimental studies suggest that collagen density alone may have effects on fibroblast behavior, including adhesion, migration, and gene expression (Loftis et al., 2003), and that furthermore, collagen density and fibroblast density play an important role in the mechanical properties of the myocardium (Fomovsky et al., 2012; van Spreeuwel et al., 2017). This multiscale model framework allows for further investigation and understanding of emergent phenomena that result from the dynamic interplay between molecular signaling, cell behavior, ECM composition, and tissue mechanics. For example, previous computational models have demonstrated that simultaneous targeting of multiple cells types rather than fibroblasts alone can enhance the efficacy of therapies for pulmonary fibrosis (Warsinske et al., 2016). Inflammatory cells, including macrophages and neutrophils, will be incorporated into a model of post-MI wound healing as the primary source and modulators of inflammatory cytokines and TGFβ input, as has been demonstrated previously in simulations of skeletal muscle and lung fibrosis (Martin et al., 2016; Warsinske et al., 2016; Virgilio et al., 2018). This will add another layer of complexity to the spatial heterogeneity of the coupled model by representing cytokine production from individual cells, diffusion of soluble cytokines and growth factors, and migration that is driven by chemokine gradients. Our goal is to develop a hybrid multiscale model that can systematically screen the effect of therapeutic interventions on the progression of cardiac fibrosis, from the level cell signaling to a tissue level of ECM remodeling, with both spatial and temporal resolution.

## Data Availability Statement

All datasets generated for this study are included in the article/Supplementary Material.

## Author Contributions

SR and TA developed the code for the coupled model. SR, AN, J-JL, SP, JH, and JS contributed to the development of model parameters and rule sets. SC and AN performed *in vitro* experiments and image analysis. SR wrote the initial draft of the manuscript. JH, SP, and JS contributed to manuscript revision. All authors read and approved the submitted version of the manuscript.

### Conflict of Interest

The authors declare that the research was conducted in the absence of any commercial or financial relationships that could be construed as a potential conflict of interest.
