# Supplementary material for: Multiscale Coupling of an Agent-Based Model of Tissue Fibrosis and a Logic-Based Model of Intracellular Signaling
Source: Front Physiol. 2019 Dec 17;10:1481. doi: 10.3389/fphys.2019.01481 (PMC6928129; doi:10.3389/fphys.2019.01481)
Supplement: Supplementary file 4 [file Data_Sheet_1.pdf]

Supplementary Material

Supplementary Figure Legends

Supplementary Figure 1. proMMP 1 expression is a combination of multiple upstream inputs. Activation patterns of network intermediates **(A)** NF-κB, **(B)** Smad3, and **(C)** AP1. **(D)** proMMP 1 network node activity level **(E)** A simplified network model representation of the immediate upstream reactions that regulate proMMP 1 expression.

Supplementary Figure 2. Coupled model produces stochastic results. Three representative images of the collagen profile at 6 weeks for simulations with migration speeds of 1 grid/10 hrs **(A-C),** 1 grid/hr **(D-E),** and 10 grids/hr **(G-I).**

Supplementary Video Legends

Supplementary Video 1. Tracking network state of individual fibroblast (from Fig. 8A). The complete network state (91 nodes) at each time step (1 hour) for the individual fibroblast tracked in Figure 8A and B.

Supplementary Video 2. Tracking network state of individual fibroblast (from Fig. 8C). The complete network state (91 nodes) at each time step (1 hour) for the individual fibroblast tracked in Figure 8C and D.

Supplementary Table Legends

Supplementary Table 1. Experimental and simulated data corresponding to Figure 10.
