# Supplementary figures and images for: Multiscale Coupling of an Agent-Based Model of Tissue Fibrosis and a Logic-Based Model of Intracellular Signaling
Source: Front Physiol. 2019 Dec 17;10:1481. doi: 10.3389/fphys.2019.01481 (PMC6928129; doi:10.3389/fphys.2019.01481)

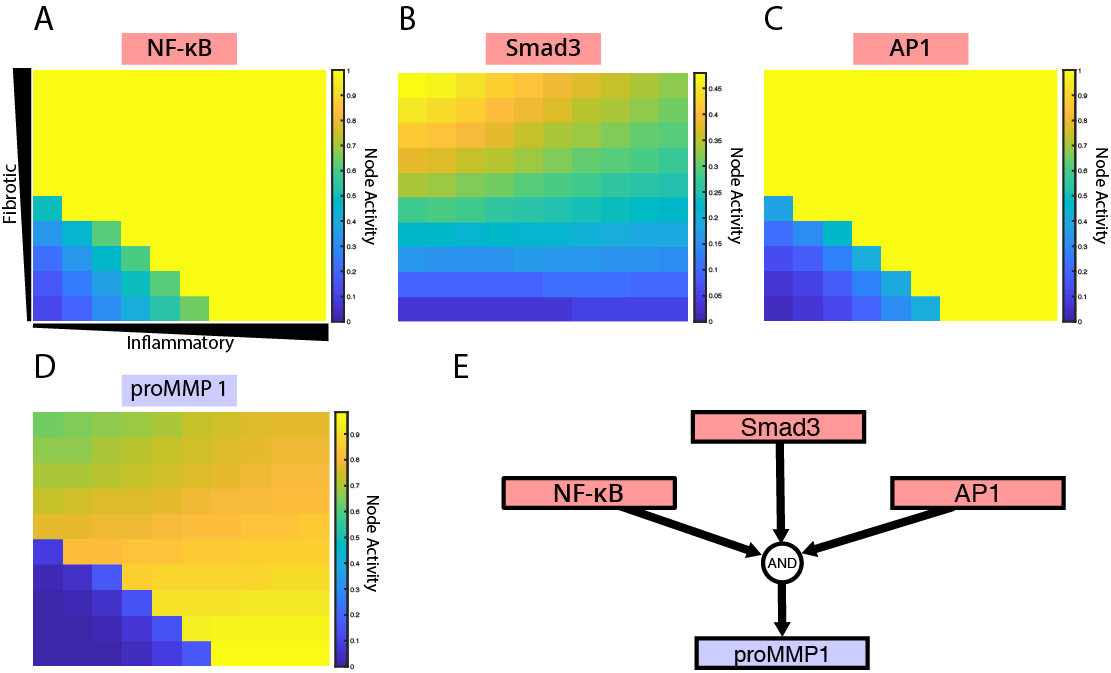

Supplement: Supplementary file 5 [file Image_1.JPEG]

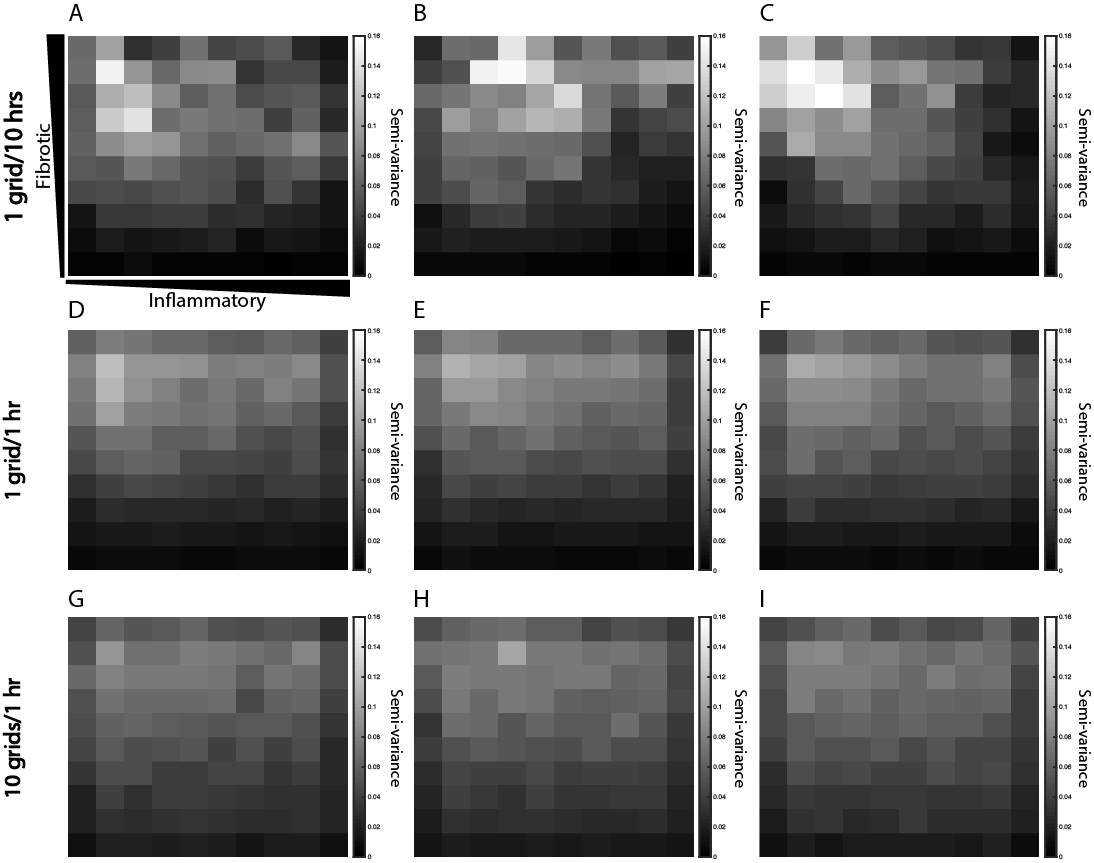

Supplement: Supplementary file 6 [file Image_2.JPEG]
